# Antibody conjugation to carboxyl-modified microspheres through N-hydroxysuccinimide chemistry for automated immunoassay applications: A general procedure

**DOI:** 10.1371/journal.pone.0218686

**Published:** 2019-06-26

**Authors:** Peter Carl, Inês I. Ramos, Marcela A. Segundo, Rudolf J. Schneider

**Affiliations:** 1 Bundesanstalt für Materialforschung und -prüfung (BAM), Berlin, Germany; 2 Department of Chemistry, Humboldt-Universität zu Berlin, Berlin, Germany; 3 LAQV, REQUIMTE, Department of Chemical Sciences, Faculty of Pharmacy, University of Porto, R Jorge Viterbo Ferreira, Porto, Portugal; 4 Technische Universität Berlin, Berlin, Germany; Consiglio Nazionale delle Ricerche, ITALY

## Abstract

Immunochemical techniques are the workhorse for sample enrichment and detection of a large variety of analytes. In contrast to classical microtiter plate-based assays, microparticles are a next generation solid support, as they promote automation of immunoassays using flow-based techniques. Antibody immobilization is a crucial step, as these reagents are expensive, and inefficient coupling can result in low sensitivities. This paper proposes a general procedure for efficient immobilization of antibodies onto TentaGel particles, via *N*-hydroxysuccinimide chemistry. The goal was the preparation of solid supports with optimum immunorecognition, while increasing the sustainability of the process. The influence of buffer composition, activation and coupling time, as well as the amount of antibody on the immobilization efficiency was investigated, resorting to fluorophore-labeled proteins and fluorescence imaging. Buffer pH and activation time are the most important parameters for efficient coupling. It is demonstrated, that the hydrolysis of *N*-hydroxysuccinimide esters occurs at similar rates as in solution, limiting the utilizable time for coupling. Finally, applicability of the generated material for automated affinity extraction is demonstrated on the mesofluidic platform lab-on-valve.

## Introduction

Enzyme-linked immunosorbent assay (ELISA) and immunochromatography are selective and sensitive techniques based on solid phases for separation, extraction and possible pre-concentration of analytes from their respective matrix, prominent examples being the detection and quantification of pharmaceuticals in the aquatic environment [1] as well as in food-stuff [2] and of biomarkers in biological fluids [3]. In contrast to batch-wise procedures, such as microtiter plate-based platforms, automated methods reduce manual handling of reagents, increasing overall precision and decreasing time-to-result [4, 5]. In this domain, microparticles have been shown to be an adequate support for carrying out immunoassays in meso- and microfluidic systems. A wide range is offered of coupling sites for biomolecules such as antibodies, combined with special anti-fouling surfaces to prevent non-specific binding while providing high compressibility for optimum fluidics. Bead-based micro-/mesofluidic immunoassays unite several advantages that turned them into a trend in immunoanalytics [6]. First, this format increases surface-to-volume ratios along with a decrease in diffusion distances, increasing the probability for antibody/antigen interaction. This results in improved sensitivity of assays and reduction in analysis time. Also, the target binders or competitors attached to the beads can be easily transported in a fluidic system using pressure-driven flow or electric fields. Another positive aspect is the large variety of molecular recognition elements available to be attached onto the beads, namely proteins, oligonucleotides, and antibodies [6–8].

The chemistry behind the conjugation of biomolecules to solid surfaces *via* activated *N*-hydroxysuccinimide (NHS) esters is commonly applied for the generation of new materials. Biomolecules are coupled to modified solid surfaces carrying carboxyl groups that were reacted previously with NHS in the presence of a carbodiimide, yielding semi-stable NHS esters. The activated esters react with amino groups present in the biomolecules, e.g. from lysine residues, to form amide bonds, compare S1 Fig [9]. During the last decade, the versatility of this coupling strategy in the production of microspheres has been demonstrated through the use of different solid materials, such as silica, [10] poly(acrylamide-*co*-acrylic acid) hydrogel, [11] and polystyrene, [12] which are intended for a wide range of applications.

Although kinetics and optimal reaction conditions are well known for NHS esters in solutions, there are few studies concerning those aspects when coupling of proteins onto microspheres occurs in suspension. In fact, even though authors often used this chemistry, they did not go through the study of reaction conditions. The lack of guidelines or standard procedures for immobilizing expensive biomolecules on solid surfaces can result in particles with low binding capacities as a consequence of insufficient coupling, and a significant waste of reagents with consequent increase in the cost of the process. These issues gain particular relevance when the final product is ought to be applied to immunoassay development as it can result in methods with low/insufficient sensitivity. In this context, the present paper proposes a standardized protocol for improving antibody immobilization on microspheres. Notwithstanding, recent literature reveals an increasing pursuit of the improvement of coupling reactions. For instance, Janissen and co-authors proposed a simple procedure to improve the bio-functionalization of glass supports with proteins and DNA using NHS chemistry [13]. Similarly, Teste et al. suggested a chemometric approach to optimize the grafting of lactalbumin on colloidal functionalized magnetic core–shell nanoparticles, using the same chemistry [14]. Another example is the optimization of the immobilization of human IgG onto Sepharose-based sorbents for immunoaffinity chromatography carried out by Mejia-Manzano and co-workers [15]. Among other techniques, fluorescence spectrometry stands out as an accurate option for evaluating coupling efficiency. For example, Koc and Alveroglu resorted to this technique to study adsorption/desorption kinetics of lysozyme from Fe_3_O_4_–polymer nanocomposites [16]. Also, researchers from the Dzantiev group determined the composition of conjugates between gold nanoparticles and proteins using the intrinsic fluorescence of the proteins [17].

As a particulate carrier, commercially available TentaGel High loaded (HL)-COOH, has frequently been used. It consists of a polystyrene core and polyethylene (PEG) groups at the surface that reduce non-specific binding (*ca*. 100 μm in diameter, when swollen). They have been applied in many areas of bioassays, e.g. as solid support for carbohydrate- [18], or peptide-based screening libraries, respectively [19], where proteins (e.g. antibodies) bind directly to surface-immobilized ligands, taking advantage of the low non-specific binding properties of the PEG anti-fouling surfaces. Furthermore, they were also used for the detection of small molecules and metals, like pesticides [20] and copper(II)-ions [21], showing the versatility of this material. Expanding the applicability of TentaGel, we studied the immobilization of proteins (i.e. antibodies) for use in automated immunoanalytical flow-based systems. The present paper proposes, after a thorough optimization of conditions, a standardized protocol for improved antibody immobilization on TentaGel microspheres using NHS ester chemistry. The aim was to prepare a TentaGel-based immunosorbent for selective analyte capture exploiting automated fluidic set-ups, while achieving cost-effective coupling optimization Coupling efficiency was assessed by laser-excitation based fluorescence imaging using fluorophore-labeled proteins as signaling units, which furthermore gave insight in specificity of binding onto these materials. Finally, anti-carbamazepine immunoglobulin G (anti-CBZ IgG) was selected as molecular recognition protein to be immobilized onto the beads and thus produce solid material for future applications, e. g. the enrichment of carbamazepine (CBZ) from environmental samples by automated immunoaffinity extraction. This pharmaceutical compound is widely employed to treat clinical seizure emergencies and as replacement therapy in epileptic patients [22], its persistence in wastewater turning it into an important anthropogenic marker and emerging pollutant [23]. Moreover, high interindividual variation is reported for CBZ metabolism, especially in co-therapy with other bioactive compounds, and thus the measurement of serum levels represents a step towards personalized medicine [24]. The feasibility of this approach was addressed at the proof-of-concept stage by assessing the binding of carbamazepine labeled with horseradish peroxidase to the decorated beads, inside the flow-based lab-on-valve (LOV) platform.

## Materials and methods

### Materials

TentaGel HL-COOH beads were obtained from Rapp Polymere (www.rapp-polymere.com). Ovalbumin (OVA), fluorescein isothiocyanate (FITC), *N*-hydroxysuccinimide (NHS), dicyclohexylcarbodiimide (DCC), dimethylformamide (DMF), 3,3’,5,5’-tetramethylbenzidine (TMB) were purchased from Sigma-Aldrich (www.sigmaaldrich.com). *N*,*N*'-disuccinimidyl carbonate (DSC) was acquired from Fluka (Buchs, Switzerland). The anti-CBZ IgG (ordering code BAM-mab 01 (CBZ)) and CBZ-HRP were produced as described elsewhere [25]. Anti-mouse IgG-Alexa647 secondary antibodies (REF: A32733, LOT: RJ243415, highly cross-adsorbed, 2 mg mL^-1^) referred to as “Alexa647 IgG”, Zeba Spin desalting micro columns and microscope slides were obtained from Thermo Scientific (www.thermofisher.com). Sephadex columns and Cy5-NHS activated ester were acquired from GE Healthcare (www.gelifesciences.com). Reagent solutions in DMF were prepared under argon atmosphere. All reactions took place at room temperature, if not stated otherwise. Ultrapure reagent water (resistivity > 18 MΩ cm) for buffer and solutions preparation was taken from a Milli-Q Reference system of Merck Millipore (www.merckmillipore.com).

### Methods

#### Preparation of OVA-FITC Conjugates

OVA (2 mg mL^-1^ in 0.1 M Na_2_CO_3,_ pH = 9.0) and FITC (1 mg mL^-1^ in DMSO) were mixed in a proportion of 1:15 (OVA:FITC). The mixture was incubated for 8 h at 4°C. Conjugates were then purified using Sephadex columns. Fractions were collected in 96-well microtiter plates, and absorbance (280 nm and 495 nm) was measured with a SpectraMaxPlus384 microtiter plate reader (Molecular Devices). Fractions presenting the highest absorbance values were recovered and stored at 4°C (0.1% (w/v) sodium azide) for further use and characterization. Concentration of the conjugate was determined with a Bradford assay using uncoupled OVA as calibrator. Fluorophore to protein ratio was measured via spectrophotometry (Nanodrop 1000, Thermo Scientific). Final concentration of the stock solution was 1.2 ± 0.12 mg mL^-1^ and F/P ratio was 1.15.

#### Preparation of Anti-CBZ IgG-Cy5 Dye Conjugates

Prior Conjugation Zeba Spin desalting micro columns were equilibrated with 3 x 300 μL of 0.13 M NaHCO_3_ (pH = 8.1). Then, 100 μL of 1.75 μg μL^-1^ anti-CBZ IgG solution was applied on the column, a stack of 15 μL of the same carbonate buffer was added and the column was eluted by centrifugation (1500 g, 2 min). To the recovered antibody fraction 235 μl of NaHCO_3_ buffer was added. To this reaction vial, 54 μL of Cy5-NHS activated ester (1 mg mL^-1^ in DMSO) were added. The vial was shaken at 1800 rpm for 2 h. The anti-CBZ IgG-Cy5 conjugate was then purified using Zeba Spin columns previously equilibrated 3 x with 300 μL phosphate buffered saline (PBS, pH = 7.6). The antibody conjugate was characterized via spectrophotometry (Nanodrop 1000, Thermo Scientific). The fluorophore to protein ratio was determined as 6.13 ± 0.25 and the concentration of the conjugate as 0.5 mg mL^-1^.

#### TentaGel beads activation and protein coupling

Prior DCC/NHS activation 5 mg of TentaGel beads (binding capacity 0.42 mmol g^-1^) were allowed to swell in 500 μL of DMF (1.00%, w/v) for 1 h. NHS and DCC stock solutions (0.5 M) were prepared in DMF. To each reaction tube, 8.4 μL—42 μL of NHS, *ca*. 1 mg of DSC and 8.4–42 μL of DCC were added, in this order. The mixture was shaken for 0.5–20 h (2000 rpm). Activated beads were divided in aliquots (5 x 100 μL, for each vial) and washed (2 x 500 μL DMF and 2 x 500 μL coupling buffer; 3 min, 10,000 *g*). Each aliquot was re-suspended in 100 μL coupling buffer. Coupling proteins were added and the reaction mixture shaken (1800 rpm) for 0.5–3 h. For coupling, PBS (pH = 7.6, see ref. [25]), NaHCO_3_ buffer (0.13 M NaHCO_3_, pH = 8.1) and TRIS buffer (pH = 8.5, please see ref. [25]) were freshly prepared.

#### Laser-excitation based fluorescence scanning

For fluorescence imaging, the beads were washed 6 x with 500 μL of PBS containing 0.1% (v/v) Tween 20 (PBS-T), and twice with 500 μL of water for salts’ removal. Afterwards the beads were spread on a cleaned and polished microscope slide and dried for 30 min at 40°C. An Axon GenePix 4300 A fluorescence microarray scanner (Molecular Devices) was used for fluorescence imaging, using a resolution of 5 μm per dot. Excitation laser (488 nm or 635 nm) intensity and photomultiplier gain were adjusted to yield the maximum signal-to-background ratio. Median fluorescence intensity was calculated for a population of 50 particles using the Software GenePix Pro7 (Molecular Devices).

#### Solid support viability for immunoaffinity applications

CBZ horseradish peroxidase (CBZ-HRP) tracer (8.3 ng mL^-1^) was incubated with 500 μg of anti-CBZ IgG beads (10 μg per 500 μg beads, activation time: 5 h, 10 eq. of DCC/NHS, coupling time: 4 h in PBS) for 120 min under orbital shaking (1800 rpm). After washing the reaction mixture 3 x with 500 μL of PBS-T and 2 x water, the beads were transferred to a microtiter plate, and 200 μL of TMB substrate solution prepared as described elsewhere was added [26]. The incubation was carried on for ten minutes, and then 100 μL of sulfuric acid (1 M) was added to stop the reaction. Absorbance was read at 450 nm with a SpectraMaxPlus384 microtiter plate reader (Molecular Devices) using 620 nm as reference wavelength. Measurements were carried out in triplicate.

#### Statistical data analysis

The paired *t*-test was applied to compare mean relative fluorescence values ± standard deviation attained for the studies related to the TentaGel beads. Non-specific binding was also calculated for all conditions tested accordingly to the Eq 1,

$$\frac{\left({FI}_{NSB}-{FI}_{AF}\right)}{{FI}_{POS\;control}}∙100\%$$ (1)

where *FI*_*NSB*_ corresponds to the fluorescence intensity of the beads after incubation with OVA-FITC or labeled IgG without previous activation, *FI*_*AutoFluorescence*_ corresponds to the autofluorescence of the beads, and *FI*_*POS control*_ corresponds to the fluorescence intensity of the beads pre-activated with DCC/NHS and incubated with OVA-FITC or labeled IgG. Relative fluorescence intensities (*RFI*) were calculated within one experimental procedure as Eq 2;

$$RFI=\frac{FI}{{FI}_{max}}∙100\%$$ (2)

where *FI* is the mean fluorescence intensity for one experiment and *FI*_*max*_ is the maximum fluorescence intensity in one experimental run.

#### Lab-on-valve immunoaffinity extraction conditions

**Fig 1:**
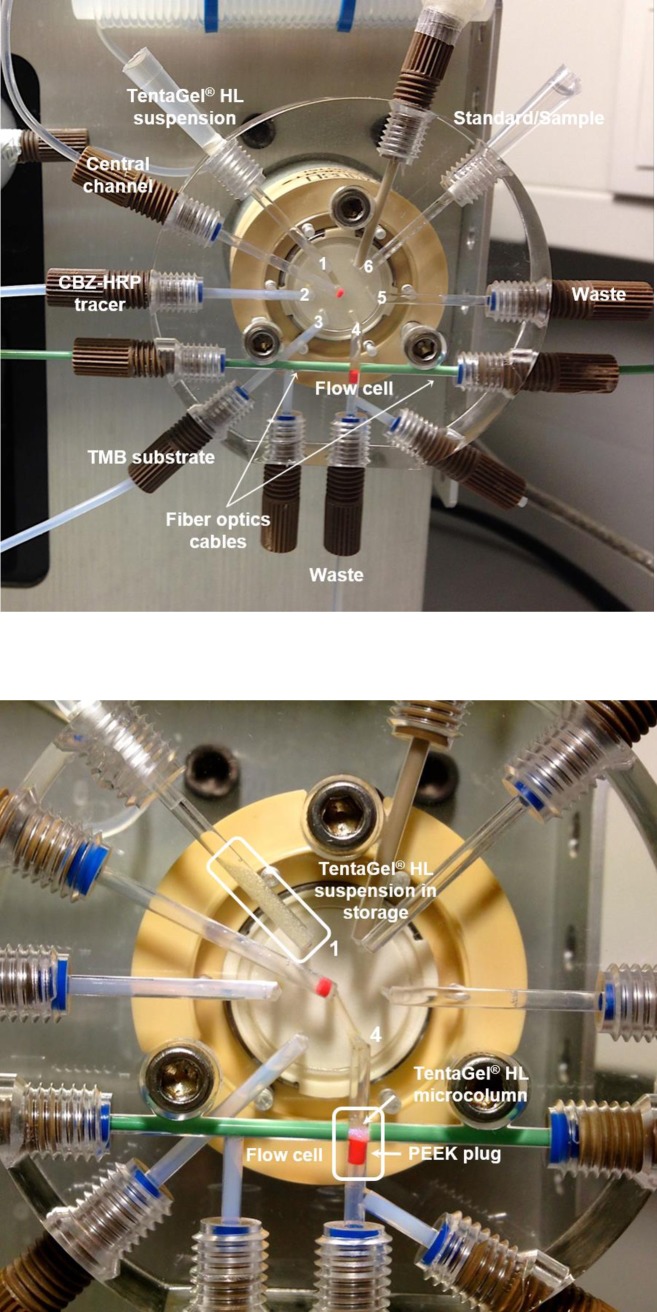
LOV device configured for micro-bead injection spectroscopy immunoaffinity extraction. A 1.0 mL syringe pump was used to aspirate and dispense reagents and carrier solution through the LOV ports (not shown for the sake of simplicity). Close-up on the LOV multiposition valve: TentaGel HL suspension in storage (port 1), and packed as immunoaffinity microcolumn inside the flow cell (port 4). A PEEK plug (0.13 mm ID, 3 mm long) was placed immediately below the light beam to retain the microspheres between the fiber optics cables while the liquid flows out. CBZ-HRP, carbamazepine-horseradish peroxidase tracer; TMB, 3,3’,5,5’-tetramethylbenzidine.

For the LOV immunoaffinity extraction of CBZ, TentaGel beads were decorated with anti-CBZ IgG using the following activation and coupling conditions: an aliquot of 500 μg of beads was activated with 10 eq. of DCC/NHS for 5 h. The beads were then washed twice with PBS-T (pH 7.4), recovered by centrifugation (18000×g, 5 min), and incubated with 1.5 μg of anti-CBZ IgG for 3 h. The beads were washed again, recovered by centrifugation (18000×g, 5 min) and transferred into the LOV reservoir (Fig 1, port 1). Detailed LOV programming routines for automated CBZ immunoextraction are presented in Table 1. The carrier stream was composed by PBS solution (pH 7.4) containing 0.15% of Tween 20. After dispersing and aspirating the beads into the central channel, 13 μL of suspension was sent to the LOV flow cell (optical path 1.6 mm, height 1.2 mm, corresponding to ca. 5 μL), and packed between two optic fibers (Fig 1, port 4). Then, the micro affinity column was perfused with a 10 or 20 ng mL^-1^ solution of CBZ labeled with horseradish peroxidase (CBZ-HRP) and the flow was stopped (90 s). After washing unbound molecules, TMB solution (0.1 mg mL^-1^) was sent to the flow cell and the flow was stopped to allow enzymatic TMB conversion (60 s), monitored on-column at 370 nm. Finally, the beads were removed by back-aspiration and discarded through the waste port (Fig 1, port 5).

**Table 1:** Automated micro-bead injection spectroscopy LOV steps for the immunoaffinity extraction of CBZ.

LOV Valve position	Flow rate (μL s^-1^)	Volume (μL)	SP Valve position^a^	Description
**Routine A) Bead column packing (52 s)**				
**-**	200	600	In	Aspirate carrier into syringe
**1**	15	10	Out	Flush and disperse solid phase
**1**	5	13	Out	Aspirate beads into the central channel
**4**	5	12	Out	Send beads to the flow cell
**5**	200	200	Out	Discard excess of beads to waste
**4**	10	392	Out	Conditioning of bead column
**Routine B) Incubation of CBZ-HRP tracer with bead column (647 s)**				
**-**	200	300	In	Aspirate carrier into syringe
**2**	5	60	Out	Aspirate CBZ-HRP solution into the central channel
**4**	1	20	Out	Send central channel content to the flow cell^b^
**4**	-	-	Out	Flow stop during 90 s^b^
**4**	1	300	Out	Wash flow cell
**Routine C) Delivery of TMB substrate (245 s)**				
**-**	200	147	In	Aspirate carrier into syringe
**4**	1	20	Out	Pack bead column
**4**	-	-	Out	Delay of 10 s; acquire reference scan
**3**	5	20	Out	Aspirate TMB solution into the central channel
**4**	1	17	Out	Start signal acquisition; send central channel content to the flow cell
**4**	-	-	Out	Flow stop during 60 s for reaction monitoring
**4**	1	130	Out	Wash flow cell; stop signal acquisition
**Routine D) Removal of bead column (18 s)**				
**-**	200	300	In	Aspirate carrier into syringe
**4**	50	100	Out	Aspirate column beads into the central channel
**2**	100	400	Out	Discard beads
**-**	200	1000	In	Aspirate carrier into syringe
	200	1000	Out	Wash flow cell

^***a***^ “Valve Out” position connects the syringe pump (SP) to the LOV central channel; “Valve In” position means that the syringe pump is connected to the carrier reservoir. **CBZ-HRP**, carbamazepine-horseradish peroxidase tracer; **TMB**, 3,3’,5,5’-tetramethylbenzidine.

^***b***^ These steps were repeated 3 times for each determination. Aspiration of reagents from LOV storage ports were followed by a delay of 3 s (steps III, VIII and XV).

## Results and discussion

### Protein immobilization onto TentaGel beads

**Fig 2:**
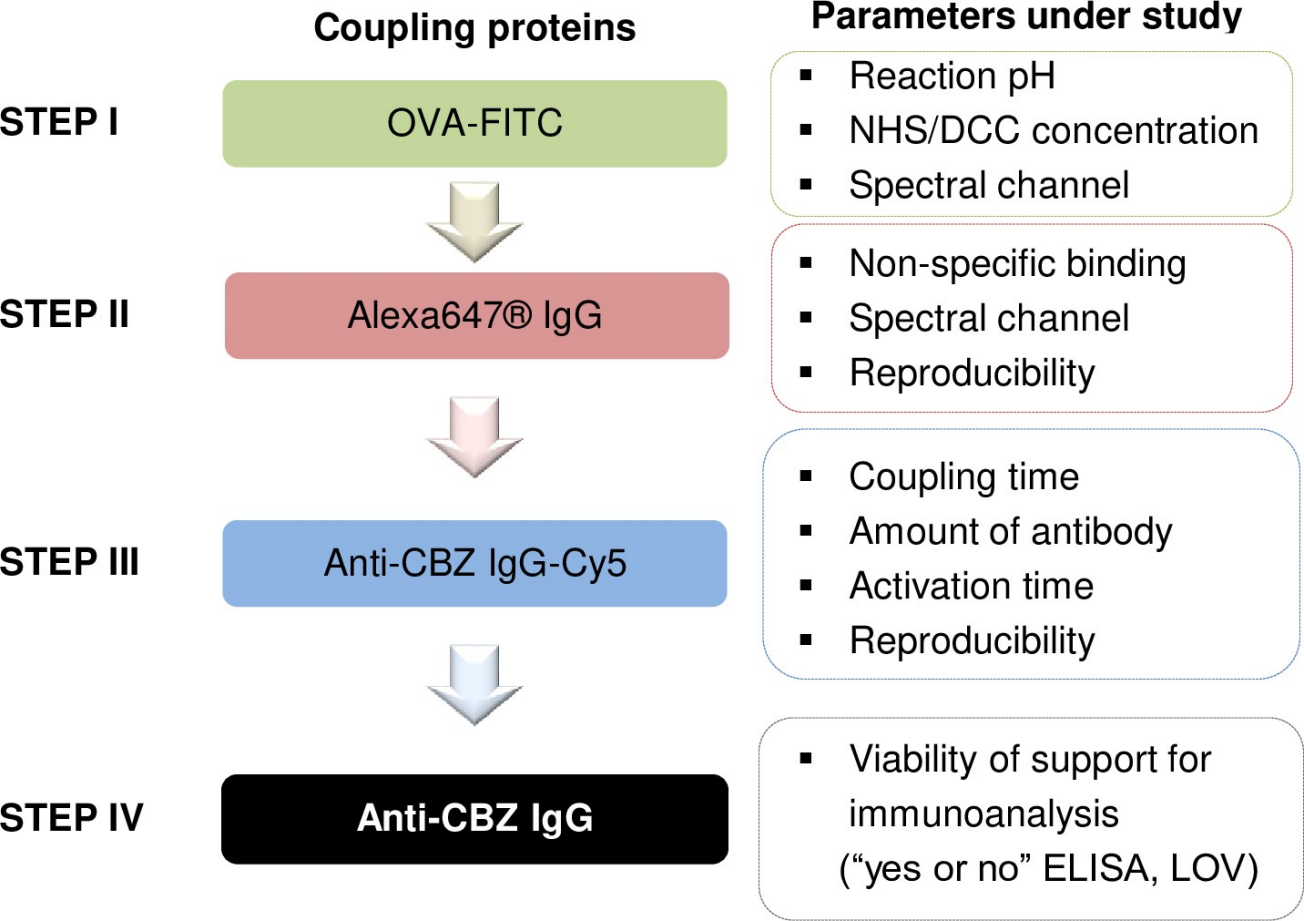
Experimental workflow for the production of anti-CBZ IgG decorated TentaGel HL-COOH beads. The applied strategy started off by the study of general reaction parameters (step I), such as the buffer system and concentration of activation agents, using the model protein OVA labeled with fluorescein (as FITC): OVA-FITC. Then the influence of more specific parameters, which depend on the final coupling target, was evaluated, namely the loading capacity of to the beads and reaction times (steps II and III). Finally, the viability of the final material for immunoassay development was tested (step IV).

NHS ester-mediated immobilization of proteins in aqueous phases is usually carried out using *N*-(3-dimethylaminopropyl)-*N′*-ethyl carbodiimide (EDC) as water-soluble carbodiimide compound in a one-pot synthesis [11, 27, 28]. However, NHS esters and activation reagents undergo fast hydrolysis, its rate being pH-dependent [29]. This narrows the window of reaction time for obtaining an exhaustive coupling. The here applied reaction scheme is depicted in S1 Fig. To circumvent this kinetically limiting factor, commercially available carboxyl-modified TentaGel beads offer the possibility of NHS activation in organic media (e.g. DMF), preventing hydrolysis before the actual coupling of the protein takes place in aqueous phase. Furthermore, the influence of activation and coupling times on protein coupling efficiency can be studied independently. Hence, an objective of this work was to establish a general procedure to improve the coupling efficiency of anti-CBZ IgG onto carboxyl-modified TentaGel beads without sacrificing a large amount of the antibody. An overview of the workflow for establishing the immobilization procedure is presented in Fig 2.

Next to basic coupling conditions (amount of activation reagents and buffer system), it was investigated, which spectral channel (“FITC channel”: Exc.: 488 nm, Em.: 510–560 nm.”Red channel”: Exc: 635 nm, Em.: 665–685 nm) is suitable for the detection of labeled proteins coupled to TentaGel beads. To evaluate the influence of the buffer system in the coupling efficiency, 1 mg beads were incubated with 100 μg of OVA-FITC conjugate in Tris buffer (pH = 8.5), carbonate buffer (NaHCO_3_; pH = 8.1) PBS; pH = 7.6 after previous activation with 10 eq. (0.42 μmol) of DCC and NHS referring to the density of COOH moieties, which were given as 42 μmol g^-1^. NHS esters are sufficiently stable to process in a two-step reaction scheme, but they will hydrolyze within hours or minutes, depending on water-content and pH of the reaction media. pH values < 7 were not tested because protein coupling reactions then occur at low rates [27, 30]. On the other hand, strong basic conditions accelerate NHS ester deactivation, which means that coupling reactions should occur under mild alkaline conditions to improve efficiency [31–33]. Immediately before scanning, buffer salts were removed from the reaction media by exchanging the solvent to water, and fluorescence intensity was recorded in the FITC channel (exc. 488 nm; em. 510–560 mm). The average particle diameter calculated for the bead population (*n* = 350) was 107 ± 8 μm. As an attempt to decrease the concentration of activation reagents, the same experiment was repeated using 2 eq. DCC/NHS.

**Fig 3:**
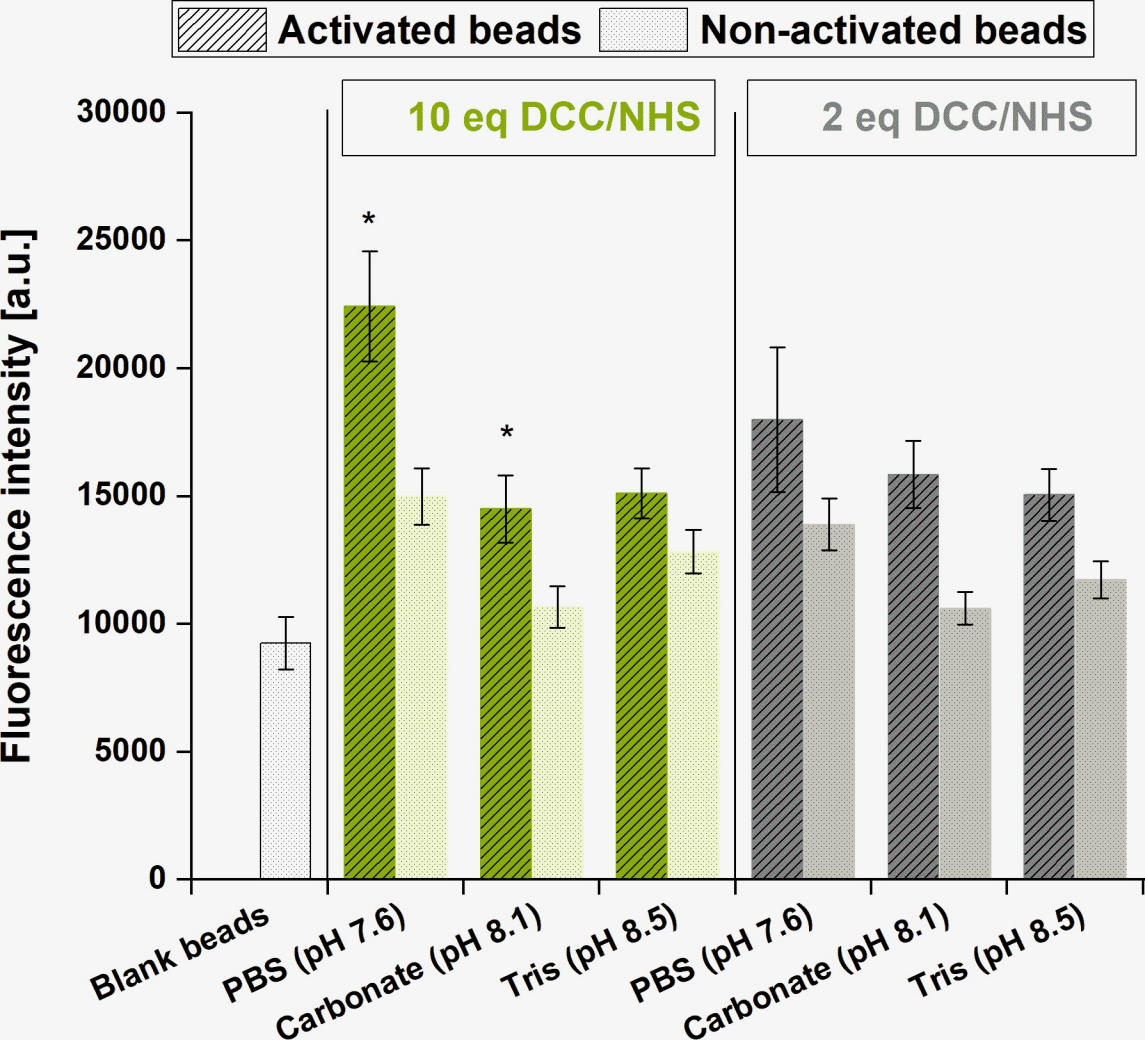
Influence of the concentration of activation reagents and buffer solution in fluorescence intensity (a.u.) obtained by coupling 100 μg OVA-FITC per 1000 μg of beads. Asterisks indicate statistically significant differences between 2 eq. and 10 eq. within each buffer system (p < 0.05). For all buffer systems, fluorescence intensity values obtained after incubating OVA-FITC to beads activated (dashed columns) with DCC/NHS and to non-activated beads (dotted columns) were statistically significantly different (p < 0.05).

Results are summarized in Fig 3. For all conditions tested, fluorescence intensity showed statistically significant differences (*p* < 0.05) between the positive (activated beads incubated with OVA-FITC) and the negative (non-activated beads incubated with OVA-FITC) batches. The lowest |t|_calculated_ value was 9.649 (t_tabulated (p = 0.05; d.f. = 98)_ = 1.984), obtained for PBS and 2 eq. of DCC/NHS. All other t-scores exceeded this value. Data analysis also revealed that the highest coupling efficiency was achieved in PBS. As for Tris buffer, there was no significant difference (*p* < 0.05) between the positive batches activated with 2 eq. and 10 eq. DCC/NHS. This result is in accordance with theoretical predictions as TRIS offers a primary NH_2_-moiety, reacting with the NHS-activated carboxylic acids, and being in large molar excess. Unexpectedly, carbonate buffer provided higher fluorescence intensities for 2 eq. DCC/NHS, showing the instability of NHS esters in strong alkaline medium and consequent unpredictability of the outcomings when this buffer system is used for coupling (*p* < 0.05). For PBS buffer, highest fluorescence intensity was obtained when beads were activated with 10 eq. DCC/NHS compared to carboxylic acid moieties. At pH = 7.6, the stability of NHS esters is increased, allowing a more efficient coupling. This was not obvious, as for other polymer-NHS ester systems, sometimes better coupling results are obtained in more alkaline buffer systems (pH = 8–9). For example, Wang et. al. explained this behavior as a consequence of amino moieties being deprotonated in such conditions, thus reacting faster with NHS esters [27], and a balance between the reaction rate of the amide bond formation and the NHS ester hydrolysis is necessary [30]. All further coupling reactions were then performed using PBS (pH = 7.6) and 10 eq. of activation reagents.

**Fig 4:**
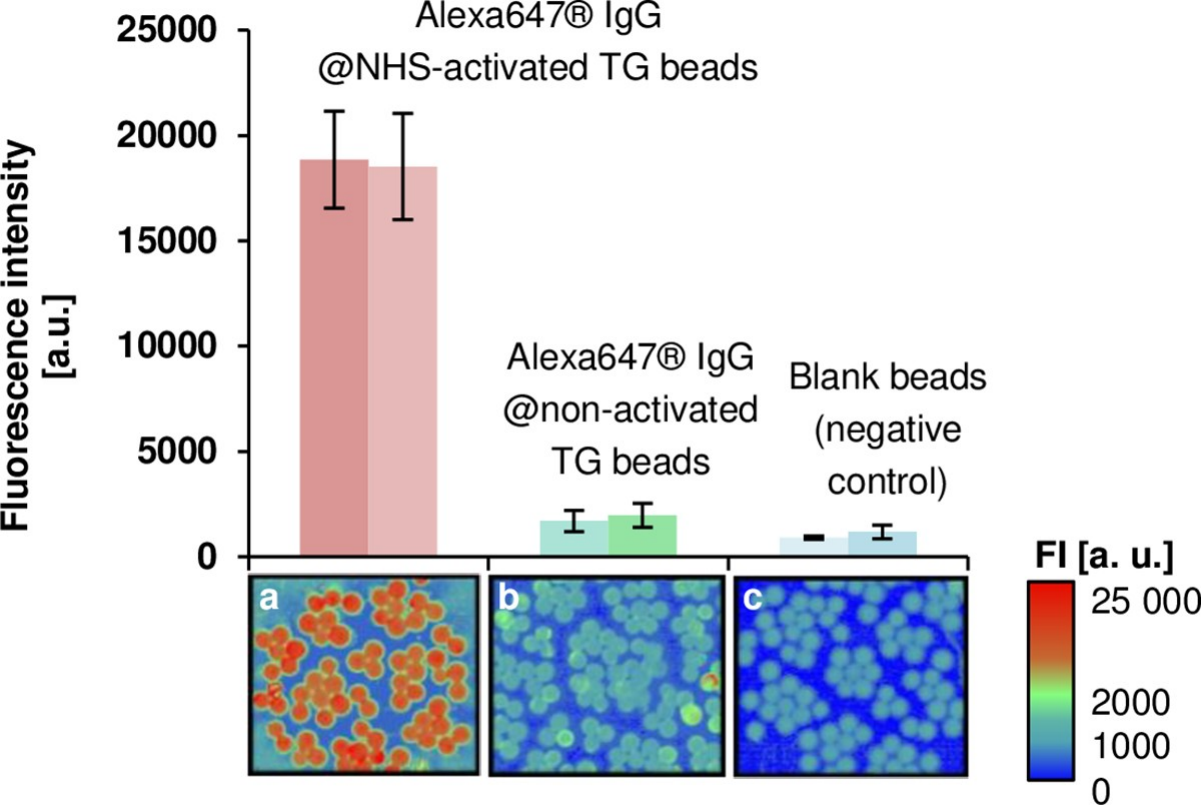
Comparison of fluorescence intensity (exc. 635 nm; em. 665–685 nm) of TentaGel (TG) beads incubated with Alexa647 IgG a) with and b) without previous NHS-activation. The mass ratio of antibody to beads was 10 μg to 500 μg. Blank beads were also scanned as negative control (c). Average fluorescence intensity values were calculated for two independent populations (*n* = 50) from the same batch of beads. The respective fluorescence scanning images are displayed below the chart (Resolution: 5μm pixel^-1^).

Despite being a suitable model for preliminary studies, the studies were not carried on with OVA-FITC because the TentaGel beads presented a relatively high autofluorescence background in the FITC channel accounting for at least 42% of the signal obtained for the protein-functionalized beads. The reason for this autofluorescence is unclear, but it is most likely due to impurities during the synthesis of TentaGel beads. Overall, values of non-specific binding were also quite high (>13% of the positive response). Hence, the model conjugate was replaced by the dye-labeled Alexa647 IgG (exc. 635 nm; em. 665–685 nm). Coupling in PBS was repeated by incubating 500 μg of beads in suspension with 10 μg of conjugate, after activation of the beads for 20 h using 10 eq. DCC/NHS. Average fluorescence intensity was calculated for two independent populations of beads (*n* = 50) to confirm the homogeneous distribution of the antibody within the bead suspension (Fig 4).

Comparing the fluorescence intensity obtained for the two independent populations of beads from the positive batch, no significant difference was observed, indicated by paired-t-test, as the |*t*|_calculated_ value was 0.682 (*t*_tabulated (*p* = 0.05; d.f. = 98)_ = 1.984). Concerning the autofluorescence of the beads, low values were observed at *λ*_*exc*_ = 635 nm. Relative fluorescence values were never higher than 0.7% compared to IgG functionalized beads under all conditions tested. Thus, a red-fluorescent dye was more suitable to study the coupling efficiency. Non-specific binding was also evaluated by incubating the beads with the same amount of labeled IgG without previous activation with DCC/NHS. Non-specific binding corresponds to twice the intrinsic fluorescence of the solid support.

**Fig 5:**
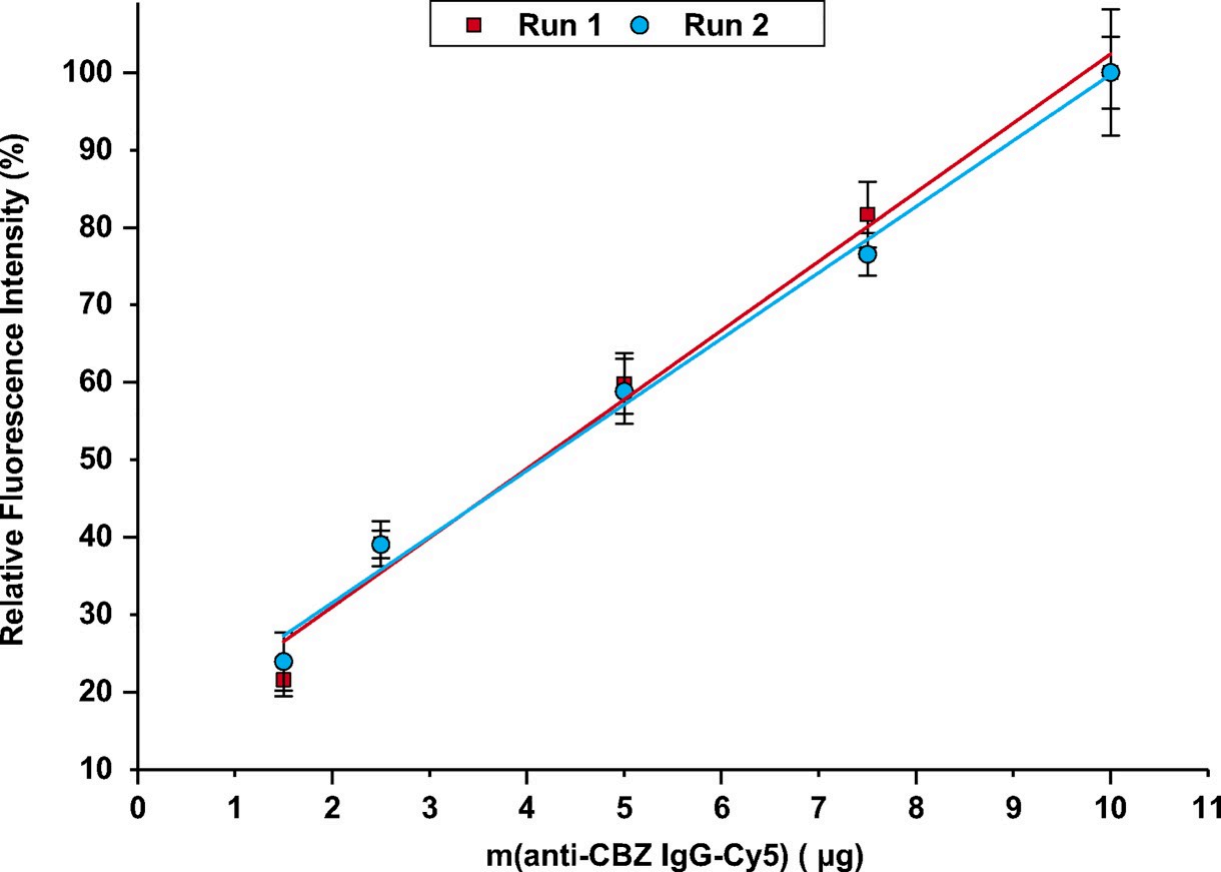
Relative fluorescence intensity (%) *versus* mass of anti-CBZ IgG-Cy5 (μg) added to 500 μg of TentaGel beads. Data were obtained from two independent runs (run one red square, run two blue dots, 20 h activation, 240 min coupling). Linear Least-Squares fit yielded a slope of 8.9% *RFI* μg^-1^ and an intercept of 13% *RFI* (R^2^ = 0.983) for Run 1, and a slope of 8.5% *RFI* μg^-1^ and an intercept of 15% *RFI* (*R^2^* = 0.990).

The next step was to perform the coupling of the anti-CBZ IgG and study activation and coupling kinetics for the target antibody. For that purpose, an anti-CBZ IgG-Cy5 conjugate was produced and coupled to the beads under the conditions previously described for the Alexa647 IgG. Cy5 was selected as dye for conjugation with anti-CBZ IgG as it possesses similar properties compared to Alexa647 with respect to absorption maxima, emission maxima, Stokes shifts, and extinction coefficients [34]. To confirm that optimal conditions obtained for OVA-FITC (coupling in PBS pH 7.6 with 10 eq. of DCC/NHS) were comparable for the anti-CBZ antibody, step 1 of the experimental workflow was repeated using the anti-CBZ IgG-Cy5 conjugate (S2 Fig). Therefore, 10 μg of the conjugate were coupled to 500 μg of TentaGel beads previously activated for 20 h with 2 or 10 eq. of DCC/NHS, in different coupling media. Similar to the results found for OVA-FITC, the use of PBS buffer and 10 eq. of DCC/NHS yielded the best coupling results, indicated by the highest fluorescence (S2 Fig). To gather insight into the loading capacity of the TentaGel beads, we investigated increasing amounts of anti-CBZ IgG-Cy5 between 1.5 μg and 10 μg. The antibodies were incubated with 500 μg of NHS-activated beads (20 h activation) for 240 min (coupling time). The relative fluorescence was plotted against the mass of anti-CBZ IgG-Cy5 added to the beads in μg per 500 μg of beads (Fig 5).

Fig 5 shows a linear dependence of the fluorescence intensity from the applied mass of antibody. Even when 10 μg of IgG were applied, a saturation of the fluorescence could not be observed. This result was further confirmed in an additional experimental run using higher concentrations of anti-CBZ IgG-Cy5 conjugate (S3 Fig). Saturation was reached when > 10 μg of anti-CBZ IgG-Cy5 per 500 μg of beads were used. This shows the high loading capacity of TentaGel beads. When used in further immunoaffinity chromatography systems or automated competitive ELISA systems, columns with tuned amount of antibody, i.e. different capacities, can be created, complying with different fields of applications. Regarding intra-assay precision, *RSD* values of fluorescence intensity values were < 16% at the low levels (1.5 μg anti-CBZ IgG-Cy5), < 7.4% for the intermediate level (5.0 μg anti-CBZ IgG- Cy5) and < 8.2% for the high level (10 μg anti-CBZ IgG-Cy5). Inter-assay precision was assessed by repeating the experiment independently on three days and calculating *RSD* values of relative fluorescence. Values were < 15% for the highest levels (7.5 μg and 10 μg), < 7.5% for the intermediate levels (2.5 μg and 5.0 μg), and < 1.0% for the lowest level (1.5 μg).

**Fig 6:**
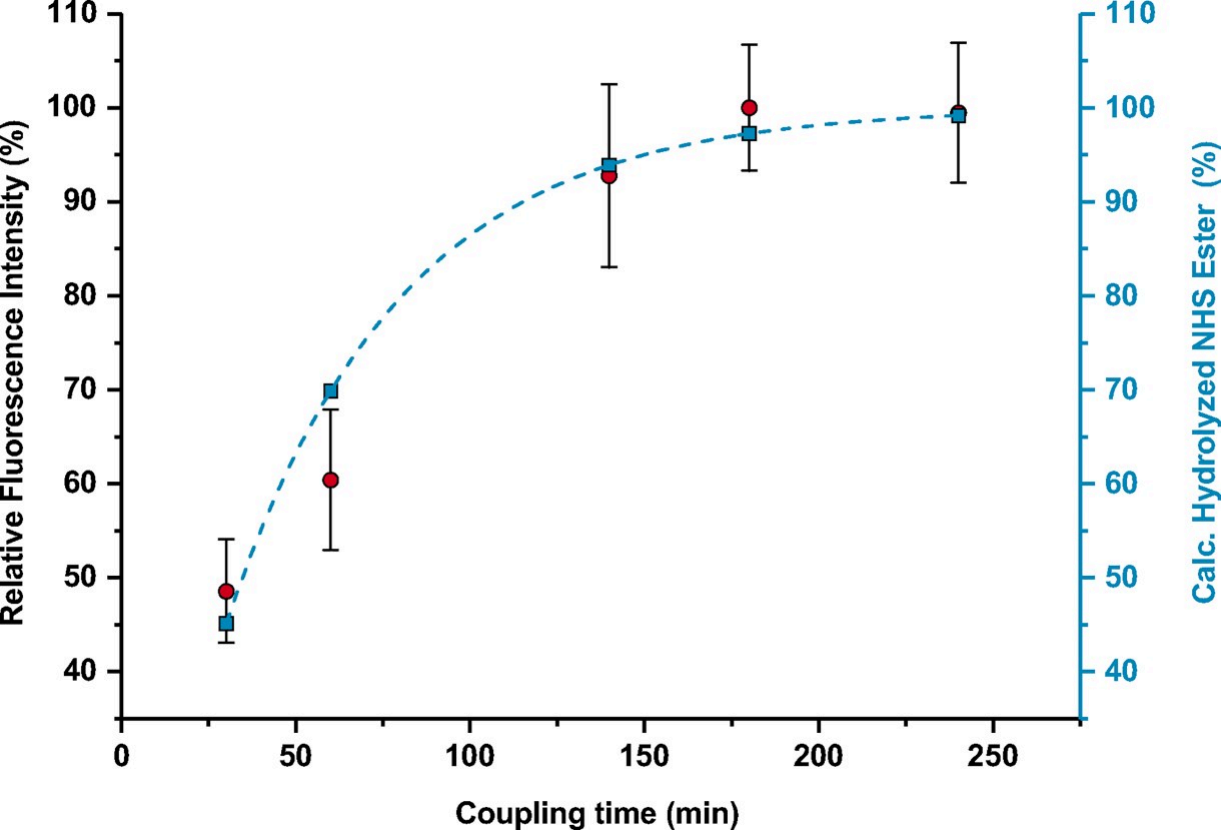
Effect of coupling time on relative fluorescence intensity (%) of the TentaGel beads. Red dots correspond to 20 h of activation with DCC/NHS and incubation with 2.5 μg anti-CBZ IgG-Cy5 conjugate per 500 μg of beads. The calculated (calc.) theoretical amount of hydrolyzed PEG-NHS ester is also presented in blue squares and dashed-line.

The time allowed for coupling reaction was also studied. As NHS esters are known to hydrolyze fast depending on the pH, the maximum reaction time between a protein and activated surfaces must be studied. For that purpose, 500 μg of NHS-activated beads were incubated with 2.5 μg of anti-CBZ IgG-Cy5 for different intervals between 30 min and 240 min. Relative fluorescence intensity values were plotted against coupling time. As comparison, the theoretical relative amount of hydrolyzed PEG-NHS ester in solution at pH = 7.60 was calculated, assuming pseudo-first order kinetics (Eq 3). It was also considered that the molecular weight of PEG polymer has no influence on the rate of hydrolysis, as reported by Pfister et al [29].

$$A_{NHS-H}=(1-e^{-k_{d}∙t})∙100\%$$ (3)

where *A*_*NHS−H*_ corresponds to the theoretical relative amount of hydrolyzed NHS ester, *k*_*d*_ to the dissociation constant of the PEG-NHS ester at 20°C and pH = 7.60 (0.02 min^-1^, extrapolated from Fig 2, Pfister et. al [29]), and *t* to coupling time in min (Fig 6).

The highest fluorescence intensities were obtained when coupling took place for more than 180 min. The mean fluorescence intensities obtained for 180 min and 240 min of coupling were not significantly different, as indicated by paired t-test (|*t*|_calculated_ = 0.368 (*t*_tabulated (*p* = 0.05; d.f = 98)_ = 1.984). It can also be observed that the amount of hydrolyzed PEG-NHS ester is related with the maximum reachable fluorescence intensity. We found that after 180 min, when > 95% of the NHS ester were hydrolyzed, no increase in the fluorescence could be obtained, as only few active NHS moieties remained accessible for protein coupling. From these results and their comparison with predicted values for NHS ester hydrolysis in solution (Fig 6), similar hydrolysis behavior was observed both in solution and on the surface of the beads, which is reported here for the first time to the best of our knowledge. Thus, the longest applicable coupling time was limited by the hydrolysis of the NHS ester.

It is important to stress that, for 180 min of coupling, the difference between fluorescence intensity values obtained for the beads incubated with a certain amount of antibody with and without previous activation corresponded to more than 91%. This means that non-specific binding accounted for less than 9% of total signal intensity.

**Fig 7:**
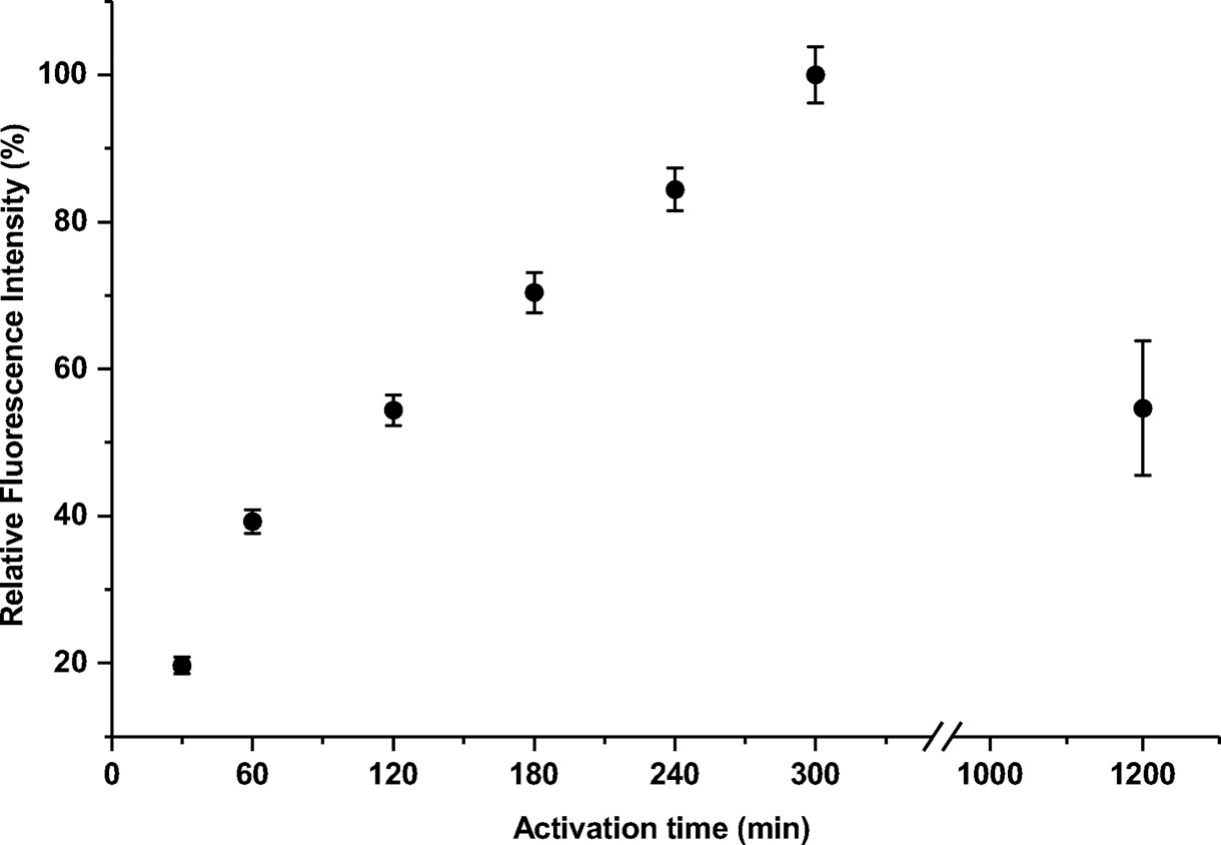
Effect of activation time on relative fluorescence intensity (%) provided by the TentaGel beads, after incubation with 5 μg anti-CBZ IgG-Cy5 conjugate per 500 μg of beads for 240 min.

Next, the influence of the activation time was studied. Before coupling, beads were incubated with DCC/NHS for different time intervals between 30 min and 20 h, setting coupling time to 240 min, and anti-CBZ IgG-Cy5 mass to 5 μg. Fluorescence intensity values are depicted in Fig 7.

In an interval between 60 min and 300 min, a linear increase of fluorescence intensity was observed. When activation time was extended to 20 h (overnight incubation), fluorescence values were only half in comparison with 300 min. Longer activation times did not necessarily improve coupling efficiency. When a carbodiimide like DCC reacts with carboxylic acids, the formation of an inactive *N*-acylurea at a slow rate has been reported, as in S1 Fig [28]. These moieties are not available to form an NHS ester, and subsequently are not accessible for protein coupling. Furthermore, it was reported that carboxylic acids on polymer-coated surfaces can form carboxylic acid anhydrides, when activated with DCC, and they will approximately react two times slower with amino groups of proteins [27]. Formation of these groups can explain the reduced fluorescence intensity observed when the activation time was extended to 20 h.

To reproduce the previous results, anti-CBZ IgG-Cy5 coupling to beads was performed under the “best” and “worst” conditions previously obtained for activation and coupling. Data confirmed that the highest coupling efficiencies can be achieved when activation and coupling time were 300 min and 180 min, respectively. Also, lowest fluorescence intensities were obtained for activation and coupling intervals of 30 min. Fluorescence intensity increased by 630 ± 52% comparing the “best” and “worst” conditions. When coupling time was extended from 30 min to 180 min, fluorescence intensity increased by 84 ± 15% (30 min activation) and 76 ± 19% (300 min activation), respectively. When the activation time was increased from 30 min to 300 min, the fluorescence intensity increased by 325 ± 49% (30 min coupling time) and 304 ± 27% (180 min coupling time). This shows, that the activation time, i.e. formation of NHS esters was more critical to the process than the coupling time.

The final step was to evaluate whether the coupled antibodies were still able to bind the target antigen, ensuring the viability of the solid support for immunoassays based on specific molecular recognition. For that purpose, a screening ELISA (positive/negative response) was performed. The beads functionalized with anti-CBZ IgG on the surface were incubated with 8.3 ng mL^-1^ of CBZ coupled to horseradish peroxidase (CBZ-HRP) for 120 min. After washing and adding 3,3’,5,5’-tetramethylbenzidine (TMB) as substrate, absorbance values at 450 nm were significantly (|*t*|_calculated_ = 4.237 (*t*_tabulated (*p* = 0.05; d.f = 4)_ = 2.776) higher (0.64 ±0.24) for the coupled beads than for the control beads (0.04 ± 0.02), showing that antibody ability of interacting specifically with the target analyte was preserved, see Table 2.

**Table 2:** Results of screening ELISA.

Experimental condition	Abs (450 nm)^a^
TentaGel beads decorated with anti-CBZ IgG (10 μg per 500μg Beads)	0.64 ± 0.24
Undecorated TentaGel beads (negative control)	0.04 ± 0.02

^a^ Mean absorbance values ± standard deviation (n = 3).

### Automated immunoaffinity extraction: Proof of concept

Mesofluidic flow systems, such as Lab-on-valve (LOV) systems, are advantageous alternatives to microchips [35]. LOV systems are considered as the third generation of flow injection systems, resulting from its downscaling by utilization of a microfabricated platform placed atop a multi-position selection valve, able to handle micro- and submicroliter volumes within channels with internal diameter of 0.5–2.0 mm. The LOV platform easily accommodates the bead injection concept [36], allowing for the manipulation of bead suspensions inside the flow conduits, including inline formation and discard of solid-phase microcolumns bearing functional groups or molecular recognition elements. This feature eliminates cross-contamination between samples and solid-phase fouling observed for other flow-based schemes.

**Fig 8:**
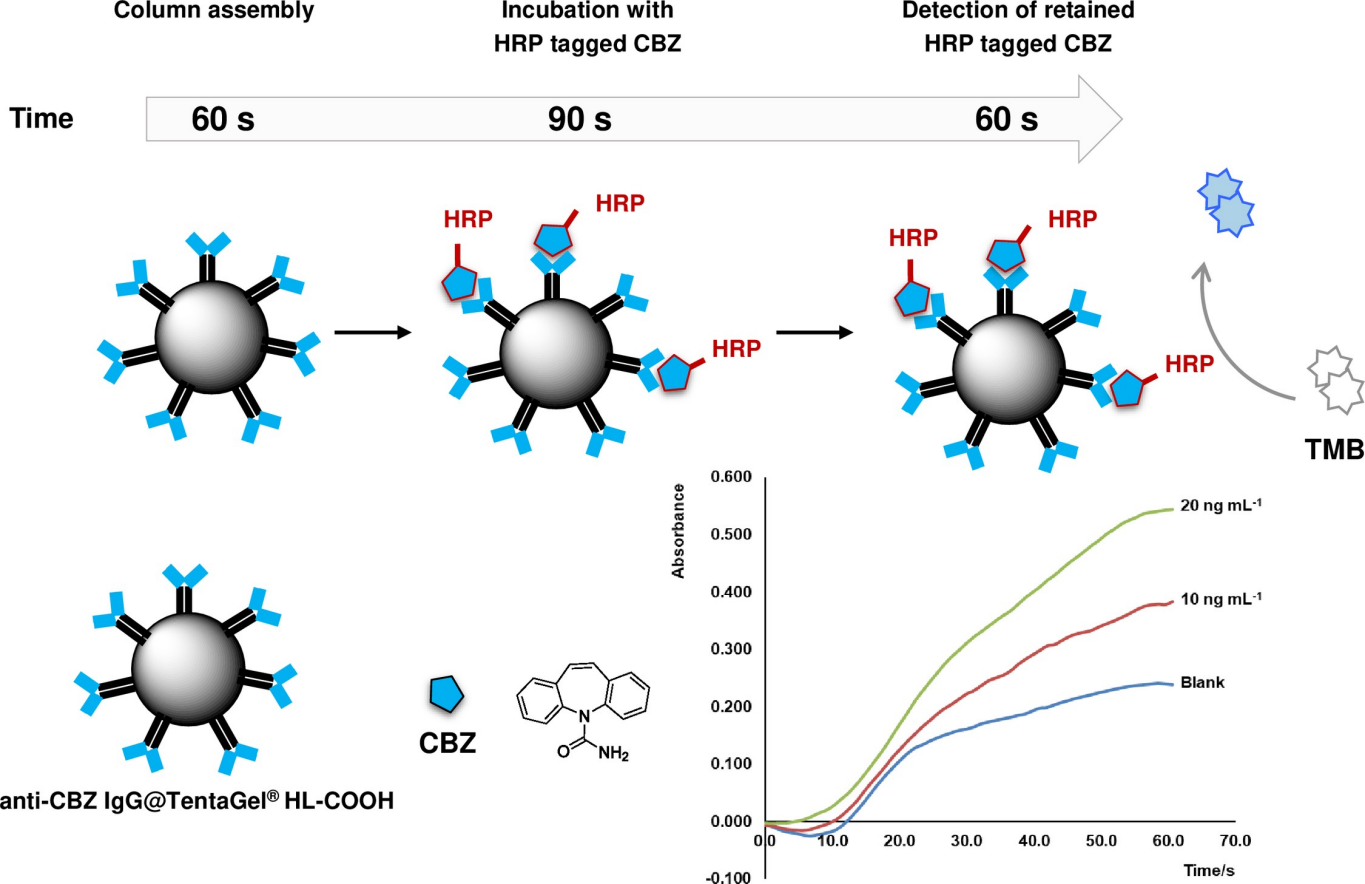
Schematic representation of the main steps of immunoextraction of carbamazepine (CBZ). Steps comprising automatic sorbent column assembly, retention of CBZ-HRP conjugate, and confirmation of CBZ immobilization by oxidation of TMB substrate (*λ* = 370 nm). HRP, horseradish peroxidase.

The proof of concept for the utilization of the developed beads was undertaken for the bead injection format detailed in Table 1. Briefly, the analytical procedure was performed automatically, under computer control of all fluidics, and it comprised packing of the bead column in front of the fiber optic CCD detector, followed by incubation with CBZ-HRP tracer and delivery of TMB substrate to reveal the binding of tracer to the solid support (Fig 8). After this assessment, the solid-phase column was automatically discarded, making the system ready for the following analytical cycle using a new sample. As represented in the absorbance *vs*. time graph (Fig 8), TMB oxidation increased according to CBZ-HRP tracer concentration, showing a measurable difference from the blank (absence of CBZ-HRP) for 10 and 20 ng mL^-1^. For instance, absorbance values taken 50 s after starting the signal acquisition were 0.220, 0.356, and 0.515 for the blank, for 10 ng mL^-1^ and for 20 ng mL^-1^ respectively.

Detection conditions in the LOV set-up are considerably different from the microplate ELISA in terms of optical path and detection wavelength (please consult experimental section), explaining the different signal outputs obtained for the blank. Despite observing a higher blank signal in the LOV set-up, which shows that TMB presents intrinsic absorbance at 370 nm, a considerable difference of signal was observed for CBZ-HRP solutions. These results show the potential for implementation of competitive ELISA schemes, targeting CBZ determination in environmental waters.

## Conclusions

This work proposes a systematic experimental approach to improve the immobilization of proteins onto carboxyl-modified microspheres for immunoassays. The study of buffer system, reagents’ concentrations, activation and coupling times on protein immobilization allowed the establishment of a standard protocol. An OVA-FITC conjugate was not a good model to perform studies of immobilization of proteins, due to its overlapping fluorescent properties and high non-specific binding. This shows the importance to choose an adequate protein-dye conjugate to perform fluorescence studies depending on the composition of the solid material. The use of fluorescent dye-labeled antibodies has proven to be a more suitable model for evaluating the effects of activation and coupling time. With our approach of creating NHS esters in water-free medium, we were able to study effects on different steps in protein immobilization. NHS esters were synthesized and consecutively coupled without the need of finding a compromise between time of activation and time of coupling. This is an advantage over applying these steps simultaneously in aqueous media via EDC/NHS chemistry. Overall, results showed that activation time, i.e. formation of NHS esters was more critical to the process than coupling time, because the applicable coupling time is limited only by the stability of the NHS esters. Also, immobilized antibodies maintained their specific recognition properties, even when long activation and coupling times were applied.

It is important to stress that the proposed protocol accounts for a decrease in production/assay costs as well as for the reduction of waste generation, complying with the principles for “green chemistry”. The establishment of the best coupling conditions leads to the production of higher quality solid phases carrying antibodies as molecular recognition elements for immunoassay development, namely flow-based affinity extraction or ELISA. This improvement provides higher sensitivities. Moreover, higher loading capacities can be achieved, yielding better material for sample extraction, namely when protein separation or pre-concentration is required. Proof of concept was successfully established for the selective retention of labeled CBZ in an automatic lab-on-valve platform, fostering future applications to environmental monitoring of trace levels of low-molecular weight pollutants.

## Supporting information

S1 Fig Reaction scheme for antibody modification of carboxyl-modified TentaGel particles and possible side reactions. Carboxylic acid groups on the surface of the beads (I) react with dicyclohexylcarbodiimide (DCC, II) to the *O-*acyl*iso*urea (III), which reacts with *N*-hydroxysuccinimide (NHS, IV) to form an NHS ester (V). The NHS ester consecutively forms an amide bond (VI) with accessible amino groups of an antibody. In a side reaction, the carboxylic acid anhydride (VII), crosslinking two carboxylic acid groups on the surface of the beads, is formed, which can react with amino groups of the antibody to yield VI, too. On further, the *O*-acyl*iso*urea (III) can undergo a rearrangement reaction to form the inactive *N*-acylurea (VIII).
(TIF)

S2 Fig Fluorescence intensity (a. u.) of TentaGel beads decorated with anti-CBZ IgG-Cy5. Data were obtained from coupling of 10 μg per 500 μg of beads. Activation and coupling times were set to 20h and 240 min, respectively. The amount of DCC/NHS (10 eq. and 2 eq.) as well as the coupling buffer system (PBS, Carbonate and Tris) were the parameters under study.
(TIF)

S3 Fig Fluorescence intensity (a. u.) versus mass of anti-CBZ IgG-Cy5 (μg) added to 500 μg of TentaGel beads. Activation and coupling times were set to 20h and 240 min, respectively. PBS was used as coupling buffer and 10 eq. DCC/NHS were used for activation. Saturation of fluorescence intensity is observed, using more than 10 μg of anti-CBZ IgG Cy5 per 500 μg of TentaGel beads.
(TIF)
