# Supplementary figures and images for: Antibody conjugation to carboxyl-modified microspheres through N-hydroxysuccinimide chemistry for automated immunoassay applications: A general procedure
Source: PLoS One. 2019 Jun 26;14(6):e0218686. doi: 10.1371/journal.pone.0218686 (PMC6594677; doi:10.1371/journal.pone.0218686)

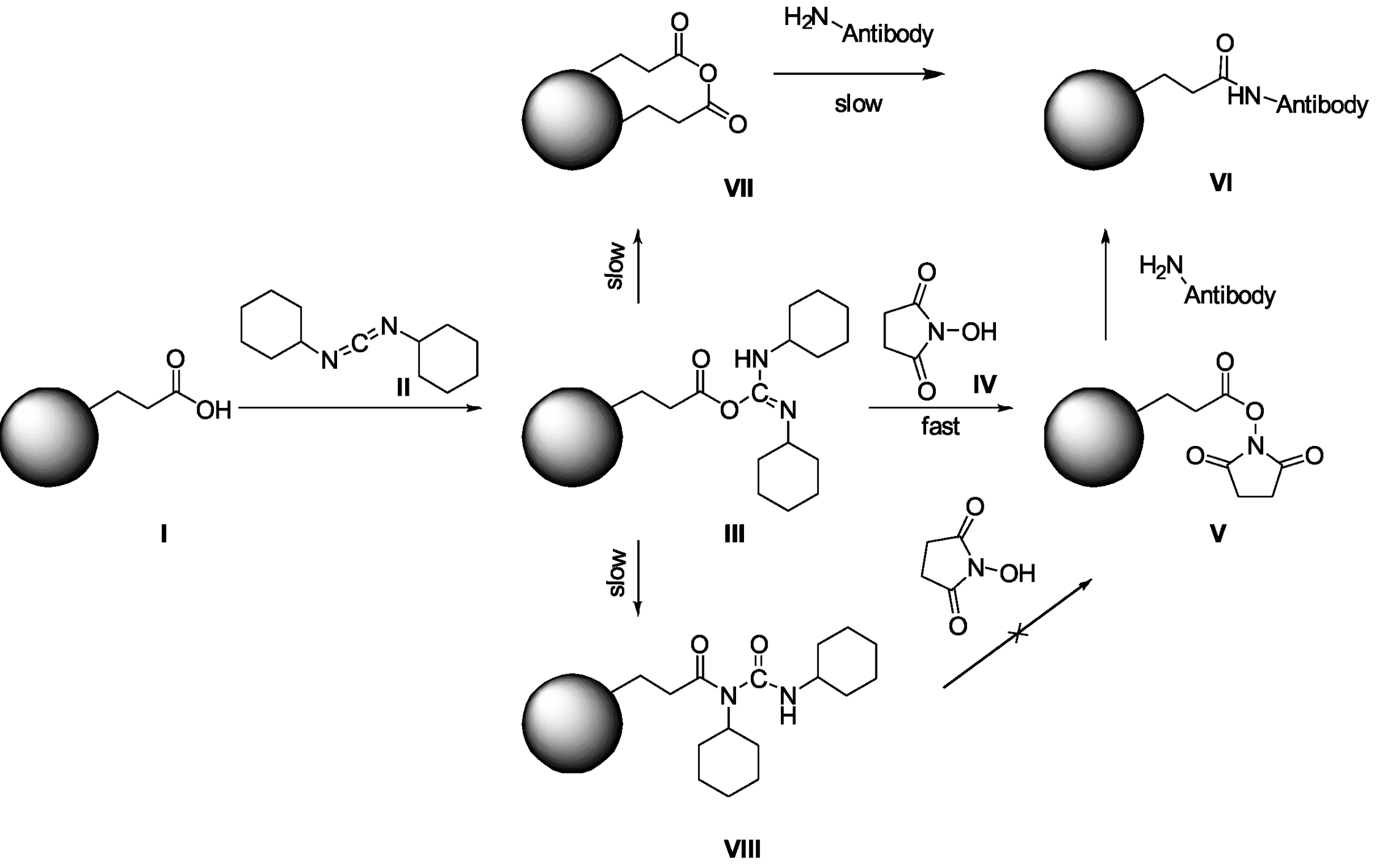

Supplement: S1 Fig — Carboxylic acid groups on the surface of the beads (I) react with dicyclohexylcarbodiimide (DCC, II) to the O-acylisourea (III), which reacts with N-hydroxysuccinimide (NHS, IV) to form an NHS ester (V). The NHS ester consecutively forms an amide bond (VI) with accessible amino groups of an antibody. In a side reaction, the carboxylic acid anhydride (VII), crosslinking two carboxylic acid groups on the surface of the beads, is formed, which can react with amino groups of the antibody to yield VI, too. On further, the O-acylisourea (III) can undergo a rearrangement reaction to form the inactive N-acylurea (VIII). (TIF) [file pone.0218686.s001.tif]

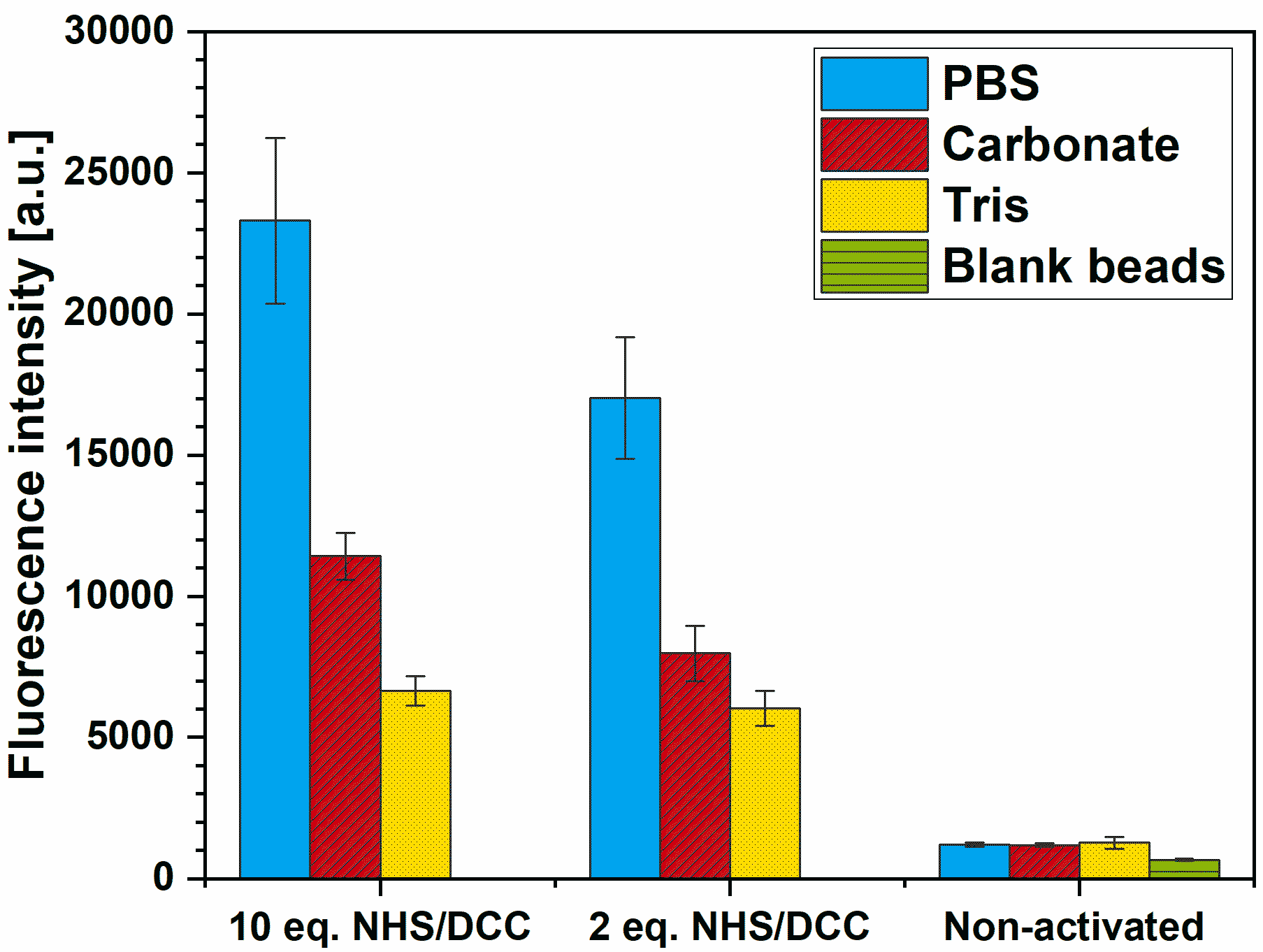

Supplement: S2 Fig — Data were obtained from coupling of 10 μg per 500 μg of beads. Activation and coupling times were set to 20h and 240 min, respectively. The amount of DCC/NHS (10 eq. and 2 eq.) as well as the coupling buffer system (PBS, Carbonate and Tris) were the parameters under study. (TIF) [file pone.0218686.s002.tif]
